# Risk factors of metabolic syndrome among hypertensive patients at Hawassa University Comprehensive Specialized Hospital, Southern Ethiopia

**DOI:** 10.1186/s12872-017-0648-5

**Published:** 2017-08-08

**Authors:** Agete Tadewos, Tariku Egeno, Antenah Amsalu

**Affiliations:** 1 Department of Medical Laboratory Sciences, Hawassa University College of Medicine and Health Science, P.O. Box 1560, Hawassa, Ethiopia; 2 Hawassa University College of Medicine and Health Science, School of Medicine, Hawasa, Ethiopia; 3 University of Gondar, College of Medicine and Health Science, School of Biomedical and Laboratory Sciences, Gondar, Ethiopia

**Keywords:** Hypertension, Metabolic syndrome, Cardiovascular disease, Southern-Ethiopia

## Abstract

**Background:**

Data regarding the prevalence of metabolic syndrome (MetS) among hypertensive patients in Ethiopia is very scarce, and the nature and the burden of MetS among these patients has not been well investigated. Therefore, the aim of this study was to assess the pattern and risk factors of MetS in hypertensive patients.

**Methods:**

A cross-sectional study was conducted at Hawassa University comprehensive specialized hospital from September 2015 to June 2016. Data on socio-demographic, clinical and anthropometric characteristics were collected from 238 hypertensive participants using WHO stepwise technique. Blood glucose and lipid profiles were determined after overnight fasting. Finally, MetS was defined according to National Cholesterol Education Program Adult Treatment Panel III Criteria.

**Results:**

The overall prevalence of MetS was 48.7% and urban dwellers had significantly higher prevalence of MetS (82.8%) compared to rural inhabitants (17.2%), *p* = 0.003. About 37.8%, 62.2%, 60.9% and 35.7% of the participants had abdominal obesity, elevated triglycerides, low HDL-c, and increased fasting blood glucose, respectively. In addition the mean HDL-c was significantly lower in MetS group compared to non-MetS group (39.4 vs.47.6), *P* < 0.0001. Age over 60 years, overweight, and obesity were associated risk factors of MetS. The adjusted odds ratio (95% CI) was 8.2 (1.1–62.4) for age over 60 years, 2.8 (1.4–5.9) for overweight and 10.7 (3.8–29.8) for obesity. Moreover monthly income of 1001–2000 Ethiopian birr, income ≥2001birr, a retirement pension, being married, divorced/widowed were also significantly associated risk factors of MetS, the adjusted odds ratio (95% CI) was 3.6 (1.1–12.5), 5.8 (1.5–22.3),5.3 (1.1–25.9),7.2 (1.4–35.9) and 16.4 (1.1–244.2), respectively.

**Conclusion:**

Metabolic syndrome is highly prevalent among hypertensive patients and this may potentiate the risk of cardiovascular problems. Therefore, regular screening of patients for individual components of MetS is vital in order to avert/limit the risks before developing cardiovascular related morbidity and mortality.

## Background

The Cardiovascular diseases (CVD) account approximately 17 million deaths worldwide in a year; it is almost one third of the total deaths [1], and 9.4 million deaths every year due to complications of hypertension (HTN) globally [2]. It is also supposed that HTN is accountable for a minimum of 45% death due to heart disease and 51% due to stroke [1]. The prevalence of non-communicable chronic diseases (NCDs) is on rising worldwide in relation to high growth rate among populations in developing countries [3]. Besides, NCDs are predicted to be highly increased in sub-Saharan Africa by 2030 [4, 5]. Of these NCDS, metabolic syndrome (MetS) is the one and it comprises a cluster of risk factors for developing CVD and which is characterized by abdominal obesity, increased blood pressure (BP), lipid derangements, hyperglycemia and insulin resistance [6–8]. The frequency of MetS in hypertensive individuals’ ranged from 30 to 40% [9]. In addition the prevalence of MetS among Ethiopian working adults was 12.5% according to National Cholesterol Education Program Adult Treatment (NCEP-ATP) III criteria [10]. However, the report from Northwest Ethiopia revealed that higher frequency of MetS (40.7%) in hypertensive patients [11].Furthermore, the use of some antihypertensive agents like diuretics or beta-adrenergic blocking drugs, may upsurge the insulin resistant state, and raises the tendency for the emerging type-II diabetes mellitus [12].

Data regarding MetS in hypertensive patients in Ethiopia is very scarce, the nature and the burden of MetS in these subjects has not been well investigated. Therefore, the aim of this study was to assess the pattern and associated factors of MetS among hypertensive individuals.

## Methods

### Study setting and study population

The study was conducted in Hawassa University comprehensive specialized Hospital, Southern Nations Nationalities and Peoples Region (SNNPR) from September 01/2015 to June 30/2016. Hawassa is the capital city of the region and located 275 km from Addis Ababa, which is the capital city of Ethiopia. This institution based cross sectional study was conducted in hypertensive patients. All hypertensive subjects age older than 18 years those who had a regular follow-up in the chronic disease clinic were eligible for the study. However, patients using lipid-altering drugs, women with confirmed pregnancy, previously diagnosed diabetic individuals, known cardiac and renal failures were excluded from the study.

### Sample size and sampling technique

The sample size was calculated based on single population proportion formula using a 95% confidence interval (CI) and 81.3% prevalence of low HDL-c among hypertensive patients according to NCEP-ATP III [11].

$$ \mathrm{n}=\frac{{\left({\mathrm{Z}}_{\mathrm{a}/2}\right)}^2\mathrm{p}\ \left(1\hbox{-} \mathrm{p}\right)}{{\mathrm{d}}^2} $$

where, P = proportion of low HDL-c, Z_α/2_ = Critical value at 95% level of confidence (Z = 1.96) d = Margin of error (5%). Based on the above formula with considering 5% non-response rate, the final sample size was calculated to be 244. To select participants from the study population, direct patient flow was checked for one week in the chronic diseases clinic and patients’ logbook was assessed additionally for its confirmation. Thus, the trend showed that the average weekly patient flow was approximately 70–80 cases for hypertensive patients. Finally, every fourth hypertensive patients were selected using systematic random sampling technique.

### Assessments and measurements

Socio-demographic, anthropometric and other relevant clinical data of the study participants were collected by pre-tested structured questionnaires. Following this, trained nurse who were working at chronic diseases clinic recorded the physical/anthropometric examinations. Blood pressure (BP) of the patients was measured using a standard adult arm cuff of mercury type sphygmomanometer after a minimum of 5–10 min rest in the clinic. The precision of the measurment was maintained by using two readings within 2–3 min differences and finally the average blood pressure (BP) was taken and recorded to assess pressure(BP) status. In addition the third measurment was taken, when the two BP readings differed by 10 mmHg within a single individual, and finally the average of three readings was used. WHO stepwise approach was applied to collect data of weight, height and waist circumference [13]. Individuals Weight and height was measured when they stood without shoes and wearing light garments. Body weight was measured using an electronic scale to the nearest 0.1 kg, and standing height was measured using a wall stadiometer to the nearest 0.1 cm. Moreover, body mass index (BMI) was calculated as the weight (in kilogram) divided by the square root of height (in meter) and it is classified according to international conventions: underweight (<18.5 Kg/m^2^), normal weight (18.5–24.9Kg/m^2^), overweight (25–29.9 kg/m^2^), and obesity (≥30 kg/m^2^) [14]. Furthermore, waist circumference (WC) was measured at the umbilicus using a non-elastic tape to the nearest 0.1 cm with patients standing erect position after the end of a normal exhalation.

### Physical activity

According to NCEP-ATP III guideline the intensity of physical activity explained as follows: *moderate* intensity *of physical activities* include: brisk walking for 30–40 min; 2-swimming-laps for 20 min; bicycling for pleasure or transportation around 5 miles in 30 min; Raking leaves for 30 min and other related activities. If an individual physical activity performance is lower than mentioned above, it is categorized under *low intensity of physical activity* [15]. In addition, *high intensity of physical activities* include: walking after work for 30 min before getting in the car/ reaching home; walking up or down 1–2 trips of stairs instead of always taking the elevator; doing daily heavy house cleaning, push a stroller/take walks with your children; or other activities which require more energy [15].

A morning 4–5 ml blood was collected from each patient after overnight fast, and then serum was obtained for biochemical tests. Serum samples were analyzed for fasting blood sugar (FBS), high density lipoprotein cholesterol (HDL-c) and triglycerides (TGs) using A25™ BioSystem chemistry analyzer [BioSystems S.A. Costa Brava 30, Barcelona (Spain) (BioSystems™, Spain)]. TGs and FBS were analyzed using enzymatic colorimetric assay method, while a quantitative determination of HDL-c was done using direct homogeneous enzymatic assay technique. All reagents used for these analytes were from Germany (Human Gesellschaftfu^¨^r Biochemica und Diagnostica mBH).

### Definition of metabolic syndrome

The presence of MetS was defined using the NCEP-ATP III guideline. The guideline illustrated that participants should have at least three of the following risk factors to be categorized under the group of MetS. This include abdominal obesity [defined as waist circumference (WC) >102 cm in men and >88 cm in women]; raised TGs level (≥150 mg/dl); low HDL-c (<40 mg/dl in men and <50 mg/dl in women); increased BP (systolic BP ≥130/diastolic BP ≥85 mmHg) and increased FBS (≥110 mg/dl) [15].

### Statistical analysis

All questionnaires were checked visually, coded and entered into EPI INFO version 3.1 and exported to Statistical Package for Social Sciences (SPSS) Version 20 for analysis. Descriptive statistics were used to describe study population in relation to relevant variables. Chi-square and or fisher’s exact test was used for categorical variables. In addition, mean differences in quantitative continuous data of the study groups was evaluated by student’s t-test. The variation in the distribution of categorical variables in study groups was assessed using univariate and multivariate binary logistic regression. Furthermore, *P*-value <0.2 was used as a cut-off to comprise variables for multivariate binary logistic regression and finally results were considered as statistically significant when the *p*-value is < 5%.

### Data quality control

Prior to actual data collection, the quality of data was assured by pre-testing of questionnaires in Sidama Zone “Bushulo” health center, which is 5 Km away from the study area. The questionnaires were evaluated for clarity and finally amendment was done after pre-testing. Socio-demographic, clinical and physical/anthropometric data of the study subjects was collected by trained nurses. In addition, the lab performance was managed daily through running quality control samples. If results fall outside established value, the run was repeated. Furthermore, the standard operating procedures manual was used to perform all the laboratory procedures.

## Results

### Socio-demographic and metabolic characteristics of the study subjects

A total of 238 hypertensive patients approached, six of them (2.5%) refused to take part in the study. Of whom 105 (44.1%) men and 133 (55.9%) were women. The mean (±SD) age of the study participants was 53.2 (14.5) years. The prevalence of Mets was 116(48.7%) and the mean age was significantly higher among MetS group when compared to non-MetS group [55.5(13.7) vs.51 (14.9); *p* = 0.02], respectively. About 73.9%, 89.9%, 36.6%, 35.3% of the study participants were urban inhabitants, married, educationally primary level and housewives, respectively. Besides urban dwellers had significantly higher MetS when compared to rural inhabitants (82.8% vs. 17.2%), *p* = 0.003, respectively. Around 11.8% had a vigorous intensity working habits in their day-to-day activities that include farming, walking long distances on foot and doing tough activities. Furthermore, 26.9% of the participants had experiences of performing physical exercises regularly and 1.7% had a history of alcoholism (Table 1).

**Table 1:** Background characteristics of hypertensive patients

Variables		Total = 238 (%)	MetS		*p-value*
			Yes = 116 (%)	No = 122 (%)	
Age, years:	Mean (±﻿SD)﻿	53.2 (14.5)	55.4 (13.7)	51 (14.9)	*0.02*
	≤30	13 (5.5)	2 (1.72)	11 (9.1)	*0.06*
	31–45	61 (25.6)	31 (26.7)	30 (24.6)	
	46–60	88 (37.0)	41 (35.3)	47 (38.5)	
	≥61	76 (31.9)	42 (36.2)	34 (27.9)	
Residence:	Rural	62 (26.1)	20 (17.2)	42 (34.4)	*0.003*
	Urban	176 (73.9)	96 (82.8)	80 (65.6)	
Marital status:	Single	18 (7.6)	5 (4.3)	13 (10.7)	*0.048*
	Married	214 (89.9)	106 (91.4)	108 (88.5)	
	Divorced/widow	6 (2.5)	5 (4.3)	1 (0.8)	
Occupation	Farmer	34 (14.3)	11 (9.5)	23 (18.9)	
	Employed	75 (31.5)	36 (31.0)	39 (32.0)	
	Housewife	84 (35.3)	49 (42.2)	35 (28.7)	
	Merchant	26 (10.9)	9 (7.8)	17 (13.9)	*0.05*
	Retirement (pension)	19 (8.0)	11 (9.5)	8 (6.6)	
Educational status:	Illiterate	58 (24.4)	25 (21.6)	33 (27.0)	
	Primary	87 (36.6)	42 (36.2)	45 (36.9)	
	Secondary	42 (17.6)	21 (18.1)	21 (17.2)	
	Tertiary	51 (21.4)	28 (24.1)	23 (18.9)	*0.67*
Income rate: in Ethiopian birr	No income	37 (15.5)	8 (6.9)	29 (23.8)	
	≤1000 birr	107 (45.0)	50 (43.1)	57 (46.7)	
	1001–2000 birr	47 (19.7)	31 (26.7)	16 (13.1)	*<0.0001*
	≥2001 birr	47 (19.7)	27 (23.3)	20 (16.4)	
Work type:	Light work	141 (59.2)	66 (56.9)	75 (61.5)	*0.43*
	Office work	69 (29.0)	38 (32.8)	31 (25.4)	
	Vigorous work^c^	28 (11.8)	12 (10.3)	16 (13.1)	
Mode of transportation	Walking/bicycle	185 (77.7)	93 (80.2)	92 (75.4)	
	Motorized vehicles	53 (22.3)	23 (19.8)	30 (24.6)	*0.37*
Intensity of physical activity:	Low	113 (47.5)	52 (44.8)	61 (50.0)	
	Moderate	90 (37.8)	43 (37.1)	47 (38.5)	
	High	35 (14.7)	21 (18.1)	14 (11.5)	*0.34*
Doing exercises regularly^b^	Yes	64 (26.9)	25 (21.6)	39 (32.0)	
	No	174 (73.4)	91 (78.4)	83 (68.0)	*0.07*
Ever drink alcohol:	Yes	3 (1.3)	2 (1.7)	1 (0.8)	
	No	235 (98.7)	114 (98.3)	121 (99.2)	*0.61* ^a^

SD, standard deviation; ^a^significance by fisher’s exact test; income in Ethiopian birr (1ETH birr = 0.044 USD);^b^patients who consistently perform any type physical exercise for a minimum of 30 min at least three times a week; ^c^patients who are working more energetic jobs like working in the factories, farm or daily laborers

### Clinical, biochemical and other features of the study subjects

About 50(21%) of the study participants had a family history of chronic illnesses (diabetes mellitus, 7.6% and hypertension, 13.4%). Majority (95%) of the participants had been using at least one anti-hypertensive agents. The range of ant-hypertensives treatment duration was two months to forty years, and 9.2% of patients were on treatment for more than ten years. In addition the mean systolic BP, diastolic BP, FBS, TGs and BMI were significantly higher in MetS group when compared to non-MetS group (*p* < 0.0001 for all). However, the mean HDL-c was significantly lower in MetS group when compared to non-MetS group (39.4 vs. 47.6), respectively, *p* < 0.0001 (Table 2).

**Table 2:** Clinical and other characteristics of hypertensive patients

Variables		Total = 238 (%)	MetS		*p-value*
			Yes = 116 (%)	No = 122 (%)	
Duration since the diagnosis of HTN	≤5 year	152 (63.9)	73 (62.9)	79 (64.8)	
	6–10 year	53 (22.3)	21 (18.1)	32 (26.2)	
	≥11 year	33 (13.9)	22 (19.0)	11 (9.0)	*0.049*
Duration since starting anti-hypertensive agents:	No	12 (5.0)	7 (6.0)	5 (4.1)	
	≤5 year	157 (66.0)	75 (64.7)	82 (67.2)	
	6–10 year	47 (19.7)	18 (15.5)	29 (23.8)	
	≥11 year	22 (9.2)	16 (13.8)	6 (4.9)	*0.055*
Family history of chronic diseases:	No	188 (79.0)	84 (72.4)	104 (85.2)	
	Diabetes	18 (7.6)	12 (10.3)	6 (4.9)	
	HTN	32 (13.4)	20 (17.2)	12 (9.8)	*0.05*
HTN drugs combination	Not started	12 (5.0)	7 (6.0)	5 (4.1)	
	Single	72 (30.3)	27 (23.3)	45 (36.9)	
	Two	129 (54.2)	69 (59.5)	60 (49.2)	
	Three	25 (10.5)	13 (11.2)	12 (9.8)	*0.14*
Mean BMI, Kg/m2, (±SD)		25.1 (5.0)	27.3 (4.3)	23.6 (4.3)	*<0.0001*
Underweight	(<18.5 Kg/m^2^)	15 (6.3)	4 (3.4)	11 (9.0)	
Normal	(18.5–24.9Kg/m^2^)	99 (41.6)	30 (25.9)	69 (56.6)	
Overweight	(25–29.9 Kg/m^2^)	83 (34.9)	49 (42.2)	34 (27.9)	
Obese	(>30 Kg/m^2^)	41 (17.2)	33 (28.4)	8 (6.6)	*<0.0001* ^a^
Mean WC, cm (±SD)		89.7 (11.9)	95.4 (11.6)	84.5 (9.8)	*0.002*
Mean SBP, mmHg (±SD)		134 (18.1)	138.7 (16.3)	131.3 (19.1)	*<0.0001*
Mean DBP, mmHg (±SD)		84.9 (12.4)	87.9 (11)	82 (12.6)	*<0.0001*
Mean FBS, mg/dl (±SD)		125.3 (50.3)	142.9 (56.9)	108.6 (36.1)	*<0.0001*
Mean HDL-c, mg/dl (±SD)		43.6 (15.2)	39.4 (14.5)	47.6 (14.9)	*<0.0001*
Mean TGs, mg/dl (±SD)		207 (121)	261 (141)	157 (65.4)	*<0.0001*

*BMI* body mass index, *DBP* diastolic blood pressure, *FBS* fasting blood sugar, *HTN* hypertension, *HDL-c* high density lipoprotein cholesterol, *SD* standard deviation, *WC* waist circumference, *SBP* systolic blood pressure, *TGs* triglycerides; ^a^significance by fisher’s exact test

### Features of metabolic syndrome and its components

**Fig. 1:**
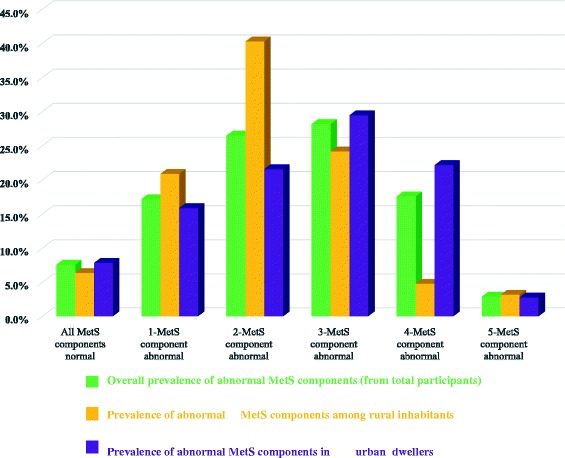
Trends of abnormal individual components of metabolic syndrome within a single individual in relation to residence

MetS was higher in women when compared to men (54.1% vs. 41.9%; *p* = 0.06), respectively. The prevalence of abdominal obesity, raised FBS, raised TGs and low HDL-c were 37.8%, 35.7%, 62.2% and 60.9%, respectively. In addition the prevalence of low HDL-c and abdominal obesity were significantly higher in women compared to men (69.9% vs. 49.5%; *p* = 0.001) and (18.1% vs. 53.4%; *p* < 0.0001), respectively. In contrast BP ≥130 mmHg was significantly higher in men (78.1%) compared to women (64.7%), *p* = 0.02 (Table 3). Furthermore, three or more abnormal components of MetS within a single individual were higher in urban dwellers compared to rural one (Fig. 1).

**Table 3:** Pattern of metabolic syndrome in hypertensive patients

Parameters	Total= 238 (%)	Men= 105 (%)	Women= 133 (%)	*p-value*
Metabolic syndrome	116 (48.7)	44 (41.9)	72 (54.1)	*0.06*
Abdominal obesity (waist circumference > 102 cm in men and >88 cm in women)	90 (37.8)	19 (18.1)	71 (53.4)	*<0.0001*
Raised triglycerides	148 (62.2)	69 (65.7)	79 (59.4)	*0.32*
Reduced HDL-c (<40 mg/dl for men and <50 mg/dl for women)	145 (60.9)	52 (49.5)	93(69.9)	*0.001*
Raised fast blood sugar (>110 mg/dl)	85 (35.7)	43 (41.0)	42 (31.6)	*0.13*
Raised SBP (>130 mmHg)	168 (70.6)	82 (78.1)	86 (64.7)	*0.02*
Raised DBP (>85 mmHg)	109 (45.8)	51(48.6)	58 (43.6)	*0.44*
Raised BP (>130/85 mmHg)	102 (42.9)	49 (46.7)	53 (39.8)	*0.29*

*HDL-c* High Density Lipoprotein-cholesterol, *DBP* diastolic blood pressure, *SBP* systolic blood pressure

### Factors associated with the presence of metabolic syndrome

Based on NCEP-ATP III criteria: being an urban, the crude odds ratio [COR (95% CI):2.3 (1.25–4.2); *p* = 0.007] and being hypertensive for more than 10 years [COR (95% CI): 2.5(1.1–5.7); *p* = 0.02] were associated with MetS. In addition, being a housewife [COR (95% CI): 3.36 (1.4–7.9); *p* = 0.006], a retirement pension [COR (95% CI):4.1(1.25–13.5); *p* = 0.02], married [COR (95% CI):2.5(1.1–7.4); *p* = 0.03], and divorced/widowed (95% CI):13(1.2–140.7); *p* = 0.02] were also associated with MetS. However multivariate analysis was maintained for possible confounding factors, being married [AOR (95% CI):7.2(1.4–35.9); *p* = 0.02], divorced/widowed [AOR (95% CI):16.4(1.1–244.2); *p* = 0.04], and a retirement pension [AOR (95% CI):5.3(1.1–25.9); *p* = 0.04] were significantly and positively associated risk factors of MetS. Furthermore, monthly income rates, age over 60 years, overweight, and obesity were also associated risk factors of MetS (Table 4).

**Table 4:** Factors associated with metabolic syndrome among hypertensive patients

Variable		MetS= Yes, n (%)	COR (95% CI)	*P-value*	AOR (95% CI)	*P-value*
Gender	Men	44 (37.9)	1.00		1.00	
	women	72 (62.1)	1.6 (0.97–2.74)	*0.06*	1.7 (0.66–4.4)	*0.27*
Residence:	Rural	20 (17.2)	1.00		1.00	
	Urban	96 (82.8)	2.3 (1.2–4.2)	*0.007*	1.3 (0.54–3.1)	*0.54*
Age in years:	≤30	2 (1.7)	1.00		1.00	
	31–45	31 (26.7)	5.7 (1.16–27.8)	*0.03*	5.8 (0.54–3.1)	*0.08*
	46–60	41 (35.3)	4.8 (1.0–22.9)	*0.05*	4.5 (0.62–32.8)	*0.14*
	≥61	42 (36.2)	6.8 (1.4–32.7)	*0.02*	8.2 (1.1–62.4)	*0.04*
Marital status:	Single	5 (4.3)	1.00		1.00	
	Married	106 (91.4)	2.5 (1.1–74)	*0.03*	7.2 (1.4–35.9)	*0.02*
	Divorced/widowed	5 (4.3)	13 (1.2–140.7)	*0.02*	16.4 (1.1–244.2)	*0.04*
Occupation:	Farmer	11 (9.5)	1.00		1.00	
	Employed	36 (31.0)	2.2 (0.93–5.26)	*0.07*	0.86 (0.24–3.07)	*0.81*
	Housewife	49 (42.2)	3.36 (1.4–7.9)	*0.006*	2.1 (0.56–7.93)	*0.27*
	Retirement pension	11 (9.5)	4.1 (1.25–13.5)	*0.02*	5.3 (1.1–25.9)	*0.04*
	Merchants	9 (7.8)	1.27 (0.42–3.8)	*0.67*	1.0 (0.22–4.76)	*0.97*
Monthly income:	No income/dependent	8 (6.9)	1.00		1.00	
	<1000 birr	50 (43.1)	2.6 (1.1–61)	*0.02*	2.2 (0.74–6.55)	*0.15*
	1001–2000 birr	31 (26.7)	6.0 (2.3–15.8)	*<0.0001*	3.6 (1.1–12.5)	*0.04*
	>2001 birr	27 (23.3)	4.2 (1.6–10.8)	*0.003*	5.8 (1.5–22.3)	*0.01*
BMI, Kg/m^2^:	Normal (18.5–24.9)	30 (25.9)	1.00		1.00	
	Underweight (<18.5)	4 (3.4)	0.57 (0.15–2.19)	*0.42*	0.89 (0.17–4.5)	*0.89*
	Overweight (25–29.9)	49 (42.2)	3.5 (1.9–6.4)	*<0.0001*	2.8 (1.4–5.9)	*0.005*
	Obese (≥30)	33 (28.4)	9.5 (3.9–22.9)	*<0.0001*	10.7 (3.8–29.8)	*<0.0001*
Duration since the diagnosis of HTN	<5 years	73 (62.1)	1.00		1.00	
	6–10 years	21 (18.1)	0.73 (0.38–1.38)	*0.33*	0.61 (0.27–1.38)	*0.24*
	>11 years	22 (19.0)	2.5 (1.1–57)	*0.02*	2.0 (0.75–5.5)	*0.16*
Doing exercises regularly:	Yes	25 (21.6)	1.00		1.00	
	No	91 (78.4)	1.7 (0.95–3.0)	*0.07*	1.9 (0.89–4.3)	*0.09*
FHCD	No	84 (72.4)	1.00		1.00	
	Diabetes	12 (10.3)	2.5 (0.89–6.8)	*0.08*	1.4 (0.36–5.4)	*0.62*
	Hypertension	20 (17.2)	2.1 (0.95–4.4)	*0.06*	2.3 (0.9–5.9)	*0.08*
Intensity of Physical activity:	Low	52 (44.8)	057 (0.26–1.22)	*0.15*	0.61 (0.22–1.74)	*0.36*
	Moderate	43 (37.1)	0.61 (0.27–1.35)	*0.22*	0.5 (0.18–1.4)	*0.19*
	High	21 (18.1)	1.00		1.00	

*AOR* adjusted odds ratio, *BMI* body mass index, *COR* crude odds ratio, *CI* Confidence Interval, *DM* diabetes, *FHCD* familial history of chronic diseases, *HTN* hypertension; income in Ethiopian birr (1ETH birr = 0.044USD)

## Discussion

MetS is a multifaceted disorder, which is responsible for a whole of cardiovascular risks and usually associated with central adiposity, dyslipidemia and insulin resistance. The overall prevalence of MetS in the study was 48.7% according to NCEP-ATP III criteria and the proportion was higher in women compared to men. Urban dwellers had significantly higher MetS when compared to rural inhabitants. In addition, abdominal obesity and low HDL-c were significantly higher in women when compared to men. Furthermore, marital status, on retirement pension, overweight and obesity were significantly associated risk factors of MetS.

A number of international studies highlight different prevalence of MetS in adult hypertensive patients like: Nigeria (42.5%), India (63.5%), Brazil (82.4%), and Iran (51.6%) [16–19], respectively. In the present study, the prevalence of MetS was 48.7% and the rate was comparable with the study report from Nigeria, which was 45.6% [20]. However, the finding was higher than the prevalence reported from Northwest Ethiopia and other African settings, which was 40.7%, 13% and 31.2% [11, 21, 22], respectively. This indicates metabolic complications of hypertensive patients bring in them to future risk of cardiovascular diseases and diabetes. The possible explanations for the variation could be genetic disparities between populations, ethnicity, socio-demographic characteristics, lifestyle, duration of hypertension and experiences of anti-hypertensive treatment. We found that the prevalence of MetS in urban inhabitants was 82.8%. This prevalence rate is higher than the report from several studies like: 35.5% in Northwest Ethiopia [11], 33.2% in Wuhan, China [23] and 27.4% in Northeast China [24]. And this suggest that a potential risk for the development of cardiovascular diseases in a significant proportion of hypertensive patients in the near future especially those patients who were living in urban. Therefore, the condition calls for a critical attention to create awareness for individuals living in urban situation concerning how to modify their life style in order to limit/prevent MetS risks.

The prevalence of MetS among hypertensive women was 54.1%. It is comparable with the prevalence reported from Nigeria, 54% [20] and other two studies of China, 52.3% [12] and 56.4% [25]. However, it is higher than the prevalence reported from Abuja state in the Nigeria, which was 38.9% [22].The differences may be attributed to the reality that only newly identified hypertensive individuals were involved in the Nigerian study. In addition, the proportion of WC was significantly higher in women when compared to men. This in line with the prevalence reported from Nigeria [20] and China [25].

We found that the prevalence of lipid profile derangement (low HDL-c) among women was 69.9%. However, high prevalence rate was reported from Northwest Ethiopia, which was 85.4% [11]. The differences in proportion may be due to the impact of age on HDL-c, as comparatively high rate (46.5%) of the study women were found to be in menopause age (>50 years) in the depicted study. Moreover, different studies reported that female sex was significantly associated risk factor of MetS [11, 12, 25]. However, this is not in line with our study finding.

We found that BMI of ≥25 (i.e. overweight and obesity) were significantly associated with the prevalence of MetS. Likewise, several studies reported that the BMI is a quantitative predictor of metabolic disturbances [12, 26]. Duration since the diagnosis of hypertension is associated with MetS [15]. In contrary, our study showed that no association between the duration of hypertension and MetS after maintaining for possible confounding factors in multivariate analysis.

Monthly income rate was significantly associated with MetS. This finding is similar with the study report from China [25]. This reflects the effect of income on MetS, however this relationship may be vary from developing country to developed one [27]. In line with the reports from Kuwait [28] and Nigeria [20], we found that age was significantly associated risk factor of MetS. In addition several studies reported that performing moderate to vigorous intensity of physical exercise decreases the developing of MetS risks [25, 29]. Concordantly we found that the low prevalence rate of MetS among patients who had an experiences of performing moderate to vigorous intensity of physical activity. Furthermore, our study indicated that marital status was significantly associated factor of MetS. This finding is similar with the reports from different studies [30–33]. Satisfaction for most individuals in marriage-like relationship might be a consequence for some abnormal components of MetS, and  also in support of our finding: the prevalence rate of obesity (a component of MetS) was higher among married subjects compared to non-married one according to Rguibi et al. report [34].

### Limitation of the study

First, our study was a cross-sectional suggesting that it cannot provide adequate evidence of causality regarding MetS and its risk factors. Second, we used only one classification was used to evaluate the frequency of MetS; a different prevalence rate might be detected if other MetS criteria like the world health organization (WHO) and or, international diabetes federation (IDF) were used. Third, our study included only hypertensive patients and that was not wide-ranged. Fourth, we did not assess the nutritional status of the individuals because of its difficulties in our situations and we have not analyzed the data of cigarette smoking because we got only one individual who had a history of smoking. Regardless of the depicted limitations, this study ultimately adds supportive information on MetS in a limited data situation of sub-Saharan Africa, mainly in Ethiopia including the study area.

## Conclusion

Metabolic syndrome is highly prevalent among hypertensive subjects. Marital status, BMI, income rate and age were found to have a significant association with MetS. This indicates the majority of hypertensive individuals are at a high risk of developing MetS. Therefore, physicians/clinicians should give attention for the problem and adhere to NCEP-ATP III guideline to diagnose patients, to manage risk conditions and to treat individuals with such metabolic complications . Furthermore, we recommend longitudinal studies to address others unexplained predictors of MetS, and to assess concealed burden of MetS in hypertensive patients at a national level.

## Abbreviations

AOR: adjusted odds ratio
BMI: body mass index
BP: blood pressure
C I: confidence interval
COR: crude odds ratio
CVD: cardiovascular diseases
FBS: fasting blood sugar
HDL-c: High density lipoprotein-cholesterol
HTN: hypertension
MetS: metabolic syndrome
NCEP-ATP: National Cholesterol Education Program-Adult Treatment Panel
SPSS: Statistical package for social sciences
TG: Triglycerides
WHO: World Health Organization
